# Advancing precision triage in strangulated small bowel obstruction: from static scores to dynamic multiparametric models

**DOI:** 10.3389/fsurg.2026.1780015

**Published:** 2026-05-18

**Authors:** Han Lin, Zhenghaoyu Huang, Mengmeng Liu, Xue Li, Yonghong Guo

**Affiliations:** 1 School of Gongli Hospital Medical Technology, University of Shanghai for Science and Technology, Shanghai, China; 2 Department of Infectious Disease, Gongli Hospital of Shanghai Pudong New Area, Shanghai, China

**Keywords:** artificial intelligence, dynamic prediction model, multiparametric integration, precision triage, risk stratification, strangulated intestinal obstruction

## Abstract

Strangulated small bowel obstruction (SSBO) is a life-threatening surgical emergency. Current clinical assessment, which predominantly relies on static and isolated parameters, often fails to accurately identify the critical transition from reversible ischemia to irreversible bowel necrosis. This diagnostic gap often results in delayed recognition and suboptimal timing of surgical intervention. Consequently, early and accurate risk stratification is imperative to guide clinical decision-making. The field is currently shifting from static evaluations toward dynamic, continuous predictive models. This narrative review examines the paradigm shift in SSBO risk assessment—from static, single-timepoint tools to integrated, intelligent systems capable of analyzing temporal data, while critically examining their current limitations in clinical validation and real-world applicability. We evaluate the characteristics and clinical applicability of various risk-stratification instruments, with a focused discussion on the role of artificial intelligence (AI) and machine learning (ML) in processing multimodal time-series data. Ultimately, we aim to outline a framework for standardized risk stratification to enhance triage precision and to advance SSBO management from a predominantly experience-based practice toward a standardized, evidence-based approach.

## 1 Introduction

Intestinal obstruction is a prevalent cause of acute abdomen requiring surgical intervention. The fundamental pathophysiological mechanism involves a mechanical blockage of the intestinal lumen, often accompanied by a compromised blood supply to the bowel wall and its mesentery (1). If not promptly identified, the condition escalates to bowel wall ischemia, necrosis, and perforation. This progression triggers diffuse peritonitis and septic shock, often culminating in death. Early and accurate recognition is therefore paramount for patient survival, yet remains clinically challenging due to nonspecific initial presentations (2).

A primary diagnostic challenge is the early differentiation of this condition, as initial clinical manifestations such as abdominal pain, distension, nausea, and vomiting are frequently indistinguishable from those of simple intestinal obstruction (3). Classical peritoneal signs (tenderness, rebound tenderness, and guarding) typically manifest only in advanced stages of bowel necrosis or perforation (4). Surgical intervention at this late stage often misses the optimal therapeutic window, resulting in significantly higher rates of bowel resection, complications, and mortality. Conversely, an excessively aggressive surgical approach poses challenges, given that approximately 70%–80% of simple intestinal obstruction cases can be effectively managed without surgery, thereby avoiding unnecessary surgical trauma and the potential for exacerbating postoperative adhesions (5). This delicate balance highlights the essential need for precise risk stratification to inform clinical decision-making, ensuring the success of conservative management while preventing the adverse outcomes associated with delayed surgery, such as bowel necrosis.

Despite notable advancements in surgical techniques and perioperative care over the past few decades, the management of SSBO continues to pose a significant clinical challenge. The clinical significance of SSBO is underscored by its rising global incidence and the substantial associated morbidity, mortality, and healthcare costs, which vary markedly across regions and populations (6–9). Numerous clinical studies have confirmed that the mortality rate associated with SSBO significantly exceeds that of simple obstruction (10–12). While the mortality rate for simple obstruction is approximately 2%, it can escalate dramatically to 25%–38% in cases of SSBO (13).

As the terminal phase of progressive mechanical obstruction, SSBO is marked by the disruption of mesenteric blood flow, with a critically limited window for intervention before irreversible damage ensues (14). Following vascular compromise due to compression, incarceration, or torsion, mucosal cell apoptosis can commence within 30 min of ischemia. Prolonged hypoxia initiates a cascade involving inflammatory cell infiltration, interstitial edema, increased capillary permeability, ultimately leading to full-thickness bowel wall necrosis, translocation of bacteria and toxins across the compromised barrier, and subsequent systemic sepsis.

Historically, SSBO risk assessment has relied on fragmented and static evaluation models. These models are insufficient to capture the dynamic progression of the disease. Traditional diagnostic approaches predominantly rely on clinical examination, laboratory tests, and imaging. Indicators such as elevated white blood cell count, serum lactate, C-reactive protein (CRP), and imaging findings like decreased bowel wall enhancement on computed tomography (CT) scans are often considered warning signs (15–17). However, clinical decisions based solely on isolated, static data present a dilemma. Overly aggressive intervention may lead to unnecessary surgery, whereas excessive conservatism can delay essential operations, resulting in severe outcomes. To address the limitations inherent in relying on single parameters, researchers have developed various predictive models and scoring systems that integrate clinical, laboratory, and radiographic parameters to enhance the accuracy of risk stratification. For example, the clinicoradiological score proposed by Schwenter et al. (18) demonstrated commendable discriminative ability (AUC=0.87); however, it remains fundamentally a static assessment based on a single time point. A prevalent limitation of such models is their conceptualization of the patient as a “snapshot in time,” rather than as a “dynamic continuum,” thereby neglecting the temporal evolution of indicators throughout the progression of the disease. In practical clinical settings, the observation period from admission to surgical decision-making can extend over several hours, during which vital signs, laboratory values, and imaging findings continuously evolve. This rich temporal dimension is one that static models fail to exploit effectively.

To address these bottlenecks, the concept of Precision Triage has emerged, drawing inspiration from the principles of precision medicine. This paradigm seeks to transition from traditional triage methods, which rely on population-level experience, to a model of continuous, dynamic risk assessment based on multi-dimensional individual data (19). Its objective extends beyond merely answering the binary question of “Is it strangulated?” to quantifying “What is the patient's probability of progressing to strangulation within a specific future timeframe?” (20). This conceptual shift is grounded in a more profound understanding of the pathophysiology of small bowel obstruction, recognizing that intestinal ischemia is a continuous, dynamically evolving process rather than an instantaneous event. Recent advancements in machine learning (ML) and artificial intelligence (AI) techniques offer the potential to integrate these temporal data streams and enable this dynamic assessment (21). However, despite their promise, most AI-based models for SSBO remain in the developmental stage, with limited external validation across diverse populations and clinical settings. Key challenges such as model generalizability, regulatory approval, and seamless integration into electronic health record systems must be addressed before these tools can reliably inform bedside decision-making.

**Figure 1:**
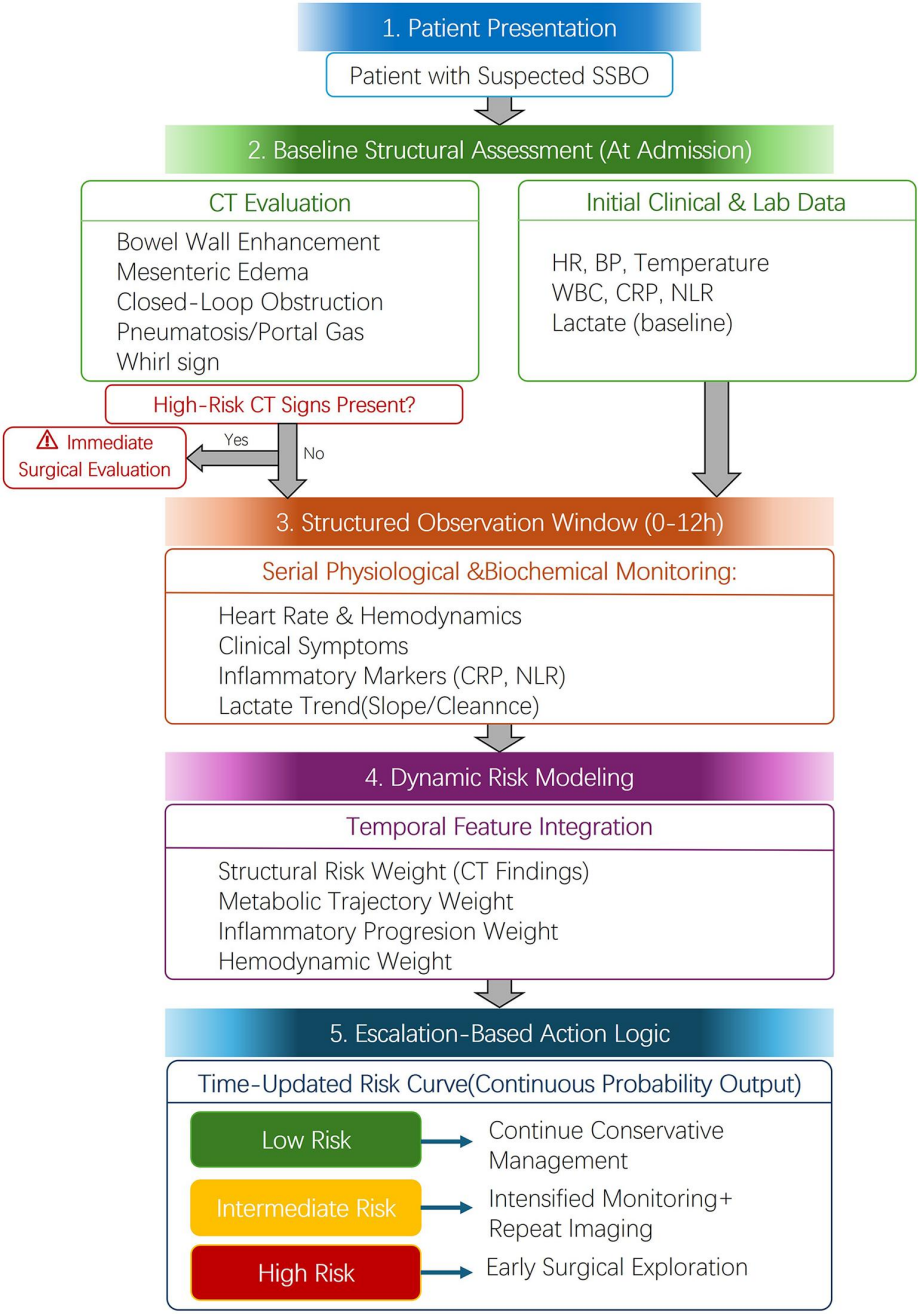
Time-dependent dynamic risk escalation framework for strangulated small bowel obstruction (SSBO). Patients without definitive high-risk CT findings enter a structured observation window during which serial physiological and biochemical data are integrated into a continuously updated probability model. Risk outputs are categorized into conceptual probability bands (low, intermediate, high) to guide escalation of care. Specific intervention thresholds are not fixed and should be prospectively validated and locally calibrated according to institutional practice patterns.

This review explores the concept of redefinition of risk assessment by considering its evolution from a static numerical output into a dynamic curve that reflects the real-time progression of disease, which may open new avenues for achieving more individualized and precise triage. In light of recent advancements reported in this domain, this narrative review aims to systematically synthesize recent key developments in dynamic multiparametric risk stratification models for SSBO. We will critically evaluate the major challenges and future directions in translating theoretical models into reliable clinical decision-support tools. Ultimately, by integrating and critically analyzing the existing literature, this article seeks to bridge the gap between computational innovation and bedside decision-making, contributing to an evolution in intestinal obstruction risk assessment from an experience-driven to a more data-driven paradigm. To illustrate this conceptual shift, we present a time-dependent dynamic risk escalation framework (Figure 1).

## 2 Temporal pathophysiological progression and monitoring basis

The lethality of SSBO arises not from a single pathological event but from a time-dependent cascade initiated by intestinal ischemia-reperfusion (I/R) injury (22). This cascade begins with transmural hypoperfusion due to mesenteric vascular compromise. When intraluminal pressure surpasses the venous pressure threshold of 20–30 cmH2O, venous return is initially obstructed. This obstruction leads to an increase in capillary hydrostatic pressure, resulting in bowel wall edema and a subsequent rise in interstitial pressure that compromises arterial inflow, ultimately culminating in transmural ischemia and hypoxia (23).

At the cellular level, this triggers a shift to anaerobic glycolysis, resulting in ATP depletion, lactate accumulation, and intracellular acidosis (24). Prolonged ischemia induces mitochondrial dysfunction and a robust inflammatory response. Damaged cells release damage-associated molecular patterns (DAMPs), and gut barrier failure allows pathogen-associated molecular patterns (PAMPs) to translocate. This cytokine expression recruits inflammatory cells, which release proteases and reactive oxygen species (ROS), further exacerbating damage to the microvascular endothelium and increasing permeability, promoting thrombosis and microcirculatory failure (25–28). Consequently, the progression from reversible ischemia to irreversible necrosis is orchestrated through a complex interplay of two major mechanisms. These include HIF-1*α*-mediated metabolic reprogramming and the inflammatory response driven by TLR4/NF-*κ*B/NLRP3 signaling.

Importantly, these pathophysiological processes correspond to clinically measurable biomarkers whose dynamic temporal patterns reflect underlying mechanisms. In the initial phase of localized ischemia, enterocyte damage results in the rapid release of Intestinal Fatty Acid-Binding Protein (I-FABP) into the bloodstream (16, 29, 30). The kinetics of I-FABP release provide early warning potential, as its levels increase sooner than traditional inflammatory markers such as IL-6 and TNF-α, and are directly correlated with the severity of intestinal ischemia (31–33). Therefore, serial monitoring of I-FABP dynamics, including the rate of increase and peak levels, is valuable for assessing baseline intestinal injury and its temporal progression (34).

Similarly, the dynamic patterns of systemic inflammation and coagulation markers—such as the doubling time of C-reactive protein (CRP), the trend of neutrophil left shift in white blood cell (WBC) count, and the kinetics of D-dimer—provide more prognostic value than isolated threshold values (35). The pathophysiological progression of SSBO occurs through three overlapping stages: reversible ischemia, the onset of an inflammatory storm, and systemic dissemination. This condition may progress to systemic inflammatory response syndrome (SIRS) and potentially to multiple organ dysfunction syndrome (MODS) (36, 37). In these advanced stages, it is crucial to closely monitor dynamic changes in vital signs and organ function indicators. Early indications of remote organ injury, such as a decreasing trend in arterial oxygen partial pressure (PaO2) (suggestive of acute lung injury) or trends indicating reduced urine output or increased creatinine levels (indicative of acute kidney injury), serve as dynamic warning signals for the risk of MODS (36, 38). Taken together, the temporal characteristics of I-FABP release, lactate trends, cytokine trajectories, coagulation indices, leukocyte dynamics, and organ function measures provide a biologically grounded, multiparametric framework linking the progression of underlying mechanisms with clinically measurable biomarkers. A summary of this multiparameter trajectory from reversible ischemia to irreversible necrosis is provided in Table 1.

**Table 1:** Pathophysiological timeline of SSBO: multiparameter trajectory from reversible ischemia to irreversible necrosis.

Time frame	0–4 h: early ischemia	4–12 h: progressive injury	12–24 h: period of transmural necrosis risk	>24 h: stage of gangrene & perforation
Molecular signaling pathways	**Hypoxia-Driven:** HIF-1*α* pathway activation initiates cellular adaptive responses (77). **Inflammatory Initiation:** Preliminary NF-*κ*B activation increases transcription of TNF-α, IL-1β (78) **Mechanosensing:** Elevated intraluminal pressure in the obstructed segment.	**Inflammatory Cascade Amplification:** Sustained NF-*κ*B activation drives massive release of IL-6 (79). **Cellular Stress & Apoptosis:** Stress kinases (e.g., p38 MAPK) trigger apoptotic programs (80). **NLRP3 Inflammasome Activation:** Caspase-1 processes pro-IL-1β and pro-IL-18.	**Mitochondrial Apoptosis:** Persistent ischemia and ROS inflict mitochondrial damage, triggering cytochrome C release and intrinsic apoptosis. **Intestinal Barrier Failure:** Marked downregulation of tight junction proteins results in complete loss of mucosal barrier integrity (81).	**Uncontrolled Systemic Inflammation:** Massive translocation of gut-derived endotoxins and bacteria fuels a fulminant, systemic inflammatory response. **Established MODS:** Widespread cellular dysfunction in remote organs, driven by persistent inflammatory mediators, hypoperfusion, and direct toxic injury.
Cellular & tissue injury	**Villous Tip Damage:** Ischemia-vulnerable epithelial cells at villous apex undergo early degeneration (82). **Functional Inhibition of Interstitial Cells of Cajal**. **Increased Mucosal Permeability** (83).	**Villous Architecture Disruption:** Extensive epithelial loss and lamina propria edema lead to villous blunting (84). **Vascular & Microcirculatory Compromise:** Pronounced congestion and dilation of submucosal vessels with significant bowel wall thickening.	**Widespread Mucosal Necrosis:** Injury progresses from villous tips to crypts, resulting in confluent mucosal necrosis. **Transmural Necrosis:** Ischemic damage extends through all bowel wall layers. Microthrombi may be evident in severe cases (44).	**Gangrene & Perforation:** Full-thickness structural disintegration and loss of bowel wall viability, culminating in perforation (85). **Peritonitis:** Extravasation of enteric contents and bacteria into the peritoneal cavity induces suppurative peritonitis.
Biomarkers	**Ultra-Early Marker:** Serum I-FABP increases sharply within 1 h, peaking around 2 h. **Local Anaerobic Metabolism:** Mild elevation in lactate.	**Fibrinolysis Activation & Microthrombosis:** Rise in D-dimer (78). **Coagulation Activation:** Increase in fibrinogen (35).	**Damage/Inflammation:** Progressive rise in C-reactive protein (CRP) (86). **Bowel Wall Ischemia:** Sustained elevation in local lactate (notably D-lactate).	**Sepsis-Associated Markers:** Marked elevation of Procalcitonin (PCT) (87). **Multi-Organ Dysfunction Indicators:** Rising creatinine, transaminases, etc., signal secondary hepatic and renal injury.
Imaging evolution	**Limited Early Direct Signs:** Findings may be limited to simple obstruction (e.g., bowel dilation, air-fluid levels). **Subtle bowel wall thickening/edema.**	**Emergence of Ischemic Features:** Bowel wall thickening (>3 mm) or thinning, increased wall density (suggesting hemorrhage), mesenteric edema/stranding, scant ascites (85). **“Stranding” or “Target” sign.**	**Findings Suspicious for Necrosis:** Pneumatosis intestinalis; portal venous gas; mesenteric venous gas; vascular interruption sign indicating occlusion (88).	**Perforation:** Free intraperitoneal air. **Peritonitis:** Significant increase in ascites, diffuse mesenteric inflammation.
Clinical presentation & signs	Symptoms: Persistent abdominal pain with colicky exacerbations, frequent vomiting. Signs: Abdominal tenderness, hyperactive bowel sounds. Peritoneal signs are often absent or equivocal.	Symptoms: Pain becomes constant and severe. Vomitus may turn bloody. Cessation of flatus and stool. Signs: Bowel sounds diminish. Unequivocal peritoneal signs (rebound tenderness, guarding) emerge.	Symptoms: Systemic toxicity appears (e.g., tachycardia, fever, confusion). Signs: Marked abdominal distension, spreading peritoneal signs, early signs of shock (e.g., cool extremities, tachycardia).	Symptoms: Septic shock, severe metabolic acidosis, overt multi-organ failure. Signs: Profound systemic deterioration, gross abdominal distension (“drum-like” abdomen), generalized rigidity/guarding, silent abdomen, hemodynamic instability requiring vasopressor support.

## 3 Performance bottlenecks of existing prediction models

### 3.1 Limitations of single-dimension models

No single parameter is sufficiently reliable for independently predicting high-risk SSBO. Clinical examination remains the primary method of assessment. Typical symptoms such as abdominal pain, vomiting, distension, and obstipation are common to both simple and strangulated obstructions. Classic signs indicative of strangulation, including fever, persistent severe pain, and peritoneal signs, often manifest only after irreversible necrosis has occurred, thereby reducing their utility for early warning. In terms of laboratory tests, inflammatory markers such as elevated white blood cell (WBC) count and C-reactive protein lack specificity. Indicators of tissue hypoperfusion and microcirculatory disturbance, including serum lactate and D-dimer, possess some diagnostic value; however, they frequently increase later than the actual onset of bowel wall ischemia, thereby reducing their sensitivity during the critical golden window for predicting strangulation (34). For instance, although serum lactate is theoretically indicative of tissue ischemia and anaerobic metabolism, numerous studies have demonstrated considerable variability in its sensitivity and specificity as an independent predictor, with the strength of evidence being limited (39, 40). Some prospective studies emphasize its potential utility; however, the proposed cut-off values are inconsistent, and the positive and negative predictive values are susceptible to confounding by various factors, thereby undermining its reliability as a standalone diagnostic marker (41).Single laboratory parameters often reflect secondary changes, such as renal impairment due to fluid loss, rather than directly quantifying gut microcirculatory compromise.

This indirect nature inherently limits their predictive performance. Imaging, particularly computed tomography (CT), is the cornerstone for diagnosing and differentiating bowel obstruction in emergency settings, exhibiting relatively high accuracy in identifying strangulation (3, 20, 42). Studies report a sensitivity of up to 72% and a specificity of approximately 86% for CT diagnosis of SSBO (43). Contrast-enhanced computed tomography findings, such as significant bowel wall thickening (>3 mm), abnormal wall enhancement (initial target sign hyperenhancement due to congestion, progressing to hypoenhancement or non-enhancement with worsening ischemia), mesenteric fat stranding, and ascites, serve as direct imaging indicators of venous congestion and inflammatory exudation (18, 44, 45). However, CT imaging provides only a static representation and may not accurately capture the dynamic changes in intestinal blood flow. The interval between imaging and surgical intervention can further influence the predictive accuracy of CT (46). Importantly, in the very early stages of strangulation, CT may not exhibit specific diagnostic signs, posing a significant challenge for early detection. Relying solely on CT angiography (CTA) for static anatomical assessment, although effective in identifying vascular occlusion, does not offer real-time insights into the metabolic state of the bowel wall or the effectiveness of collateral circulation, and it carries inherent risks associated with contrast agents.

These unidimensional models oversimplify a complex, multifactorial pathological process. Their primary flaw lies in conceptualizing patients as “snapshots in time” rather than dynamic continuums, thus ignoring critical temporal trends. In response, recent investigations now explore novel composite inflammatory indices. Yet, the absence of standardized development and validation protocols continues to hamper model comparability and clinical utility.

### 3.2 Advancements and limitations of multiparametric static models

In addressing the constraints of one-dimensional assessments, researchers have sought to develop multiparametric static models that integrate clinical features, laboratory indices, and imaging findings (18). These models are generally constructed using retrospective data and employ statistical techniques such as multivariable logistic regression or LASSO regression to identify independent risk factors and develop scoring systems, thereby enhancing predictive accuracy and robustness. The key characteristics, performance, and limitations of the major static prediction models discussed are compiled in Table 2 for a comparative overview.

**Table 2:** A compendium of published risk prediction models for strangulated small bowel obstruction.

Scoring system	Core predictors	Methodology/technical characteristics	Predictive performance	Key advantages	Primary limitations	Recommended application context
I-FABP Dynamic Monitoring (Wu et al., 2021) (34)	Serum I-FABP concentration	Meta-analysis (8 studies) evaluating a single biomarker's diagnostic utility for intestinal ischemia.	AUC=0.83 Sens=0.75, Spec=0.83 Diagnostic OR = 14.19	Rapid & Simple: Ideal for point-of-care testing. Early Signal: Reflects mucosal injury earlier than conventional markers.	**Significant inter-study heterogeneity.** Moderate Sensitivity: Risk of false negatives limits standalone use.	Adjunctive screening in the ED to raise early suspicion; must be integrated into a comprehensive diagnostic workup.
SIRS Clinical Model (Tsumura et al., 2004) (37)	1. ≥2 SIRS criteria^a^ 2. Abdominal rigidity on exam	Simplified clinical model. Significant univariate predictors confirmed by multivariate logistic regression.	**Multivariate Analysis Results:** SIRS presence: OR=14.30 Abdominal rigidity: OR=5.87 **Univariate:** Age, gender, WBC also significant.	Accessible: Utilizes universally available vital signs and exam findings.	Single-center, retrospective, small sample. Subjective assessment of rigidity; lacks robust validation.	Preliminary emergency screening, suitable for resource-limited settings to rapidly identify high-risk patients when combined with clinical assessment.
BAR-N Score (Zheng et al., 2025) (21)	4 variables: 1. Rebound tenderness (binary) 2. BMI (continuous, log-transformed) 3. Neutrophil percentage (*N*, continuous) 4. AST (log-transformed)	Clinical-lab model. LASSO regression for feature selection from 28 candidate variables. Multivariable logistic regression for model building. Retrospective single-center study (*n* = 453).	Validation cohort: AUC=0.750 (95%CI: 0.65–0.85) Specificity=90.3% Sensitivity=31.8% Accuracy=80.7% NPV=87.2% PPV=38.9% Training AUC=0.784	Practical & Low-Cost: Leverages routine admission data. High NPV (87.2%): Effective for ruling out low-risk patients.	Poor Sensitivity: Unsuitable for ruling in disease or for definitive diagnosis. Lacks imaging data and external validation.	Preliminary emergency screening, serves as an auxiliary tool prior to imaging, especially for rapidly excluding low-risk patients in resource-limited contexts.
CT Dual-Threshold Model (Chai et al.,2022) (48)	Objective HU measurements: 1. Enhancement value (post- minus pre-contrast HU difference). 2. Non-contrast density increase (HU difference between abnormal and normal bowel wall on non-contrast scan).	Quantitative CT model. ROC-derived optimal thresholds for distinguishing necrosis from ischemia.	Diagnosing Necrosis: Enhancement value < 21.5 HU (AUC 0.925) OR Non-contrast density increase > 16.5 HU. High specificity.	Objective & Reproducible: Minimizes reader subjectivity. High Discriminatory Power: Effectively identifies necrotic bowel requiring resection.	**Requires a dedicated contrast-enhanced CT protocol**. **Manual measurement adds time and potential for error**.	Adhesive SBO patients who have undergone non-contrast+contrast CT, assisting in determining the need for bowel resection.
Schwenter Clinico-Radiological Score (Schwenter et al.,2010) (18)	6 items (1 point each): 1. Pain duration ≥4 days 2. Abdominal guarding 3. CRP ≥75 mg/L 4.WBC ≥10 × 10⁹/L 5. CT ascites ≥500 mL 6. CT reduced bowel wall contrast enhancement	Prospective single-center cohort study (2004–2007). *n* = 233 consecutive SBO episodes. Multivariate logistic regression with 10-fold cross-validation. Score calculated as simple additive sum (0–6).	AUC=0.87 (95%CI: 0.79–0.95) Cross-validated AUC=0.84 (95%CI: 0.76–0.92) Score ≥3: Sens 67.7%, Spec 90.8%; Score ≥4: 100% required bowel resection (12/12); Score 0–1: 6% required resection (4/65)	**Comprehensive & Early:** Pioneered systematic multi-parameter integration. **Clinically Friendly:** Simple additive score.	**Indeterminate Range:** Scores of 2–3 pose a management dilemma. **Dependent on contrast-enhanced CT.**	Risk stratification for SBO patients with available contrast CT, aiding in the decision for operative vs. non-operative management.
Huang Clinico-Radiological Score (Huang et al., 2018) (39)	5 items (variable points): 1. Temp ≥38.0 °C (78 pts) 2. Peritoneal irritation sign (112 pts) 3. WBC >10 × 10⁹/L (65 pts) 4. CT: ascites (122 pts) 5. CT: thick-walled bowel ≥3 mm (104 pts)	Multivariate logistic regression model integrating clinical, laboratory, and CT parameters. Retrospective single-center study (*n* = 417). Score calculated as: Y = 100 × log(OR)	AUC=0.935 (95%CI: 0.900–0.969) Optimal cutoff: 132.5 pts Sens=85.5%, Spec=88.4% Score ≥299: Sens 40.8%, Spec 100%	Excellent Discrimination: Very high AUC indicates strong predictive ability. Direct Risk Stratification: Score correlates with probability of strangulation.	Single-center retrospective design. Peritoneal sign assessment is subjective and variable.	Comprehensive risk assessment for hospitalized SBO patients with full CT and lab workup, to guide treatment pathway (operative vs. non-operative). **Note:** Score >132.5 suggests high risk for SSBO.
Antoine-Béclère Score (ABS) (Maraux et al., 2022) (47)	3 items (1 point each): 1. Age-adjusted Charlson Comorbidity Index ≥4 2. CT-confirmed distal (ileal) obstruction 3. Small bowel max diameter/anteroposterior abdominal diameter ratio >0.34	Clinico-radiological model. Retrospective derivation (*n* = 171)+prospective validation (*n* = 72) at single center. Designed specifically for uncomplicated adhesive SBO.	AUC=0.69 (95%CI: 0.61–0.77) Score ≥2: Sens 75.3%, Spec 57.1% Risk stratification: Score 0–1: 21% surgical risk; Score 2–3: >50% surgical risk	**Ultra-Rapid Triage:** All parameters obtainable at admission in <10 min. **Morphological Innovation: “**Bowel/abdominal diameter ratio” accounts for individual anatomic variation.	Moderate Performance: AUC 0.69. Limited Validation: Single-center validation. Scenario-Specific: Applicable only to uncomplicated ASBO without emergent surgical indications.	Rapid triage at emergency department intake to filter uncomplicated ASBO patients requiring early surgery.
High-Risk Transmural Necrosis Model (Xu et al., 2022) (40)	7 Independent Risk Factors: 1. Pain duration ≤ 3 days 2. Rebound tenderness 3. Diminished/absent bowel sounds 4. Hypokalemia 5. Hyponatremia 6. Elevated Blood Urea Nitrogen (BUN) 7. High imaging score (CT-based)	Multidimensional integrated model. Retrospective training (*n* = 281) with prospective validation (*n* = 80). Binary logistic regression with risk score formula: RS = 1.328 × Pd + 1.649 × Rt + 1.611 × Bs + 1.307 × K + 1.323 × Na + 1.470 × BUN+2.422 × Rad−6.009.	AUC=0.857 (95%CI: 0.793–0.920) [Training] AUC=0.910 (95%CI: 0.843–0.976) [Validation] Risk stratification: Low-risk (≤−3.091): 1% StBO rate; Medium-risk (−3.091 < to ≤ −1.472): 20% StBO rate; High-risk (>−1.472): 45% StBO rate	**Superior discrimination:** Validation AUC 0.910 outperforms radiology-only models. **Necrosis prediction:** High-risk group shows 24% transmural necrosis vs. 0% in low-risk group. **Clinical triage:** Clear 3-tier risk stratification directly guides management decisions.	Single-center internal validation: Prospective validation limited to same institution. Complex calculation: Requires formula computation, not point-of-care friendly. Subjective signs: Rebound tenderness and bowel sounds assessment have inter-observer variability.	**Early admission risk assessment (within 24 h)** for SBO patients with complete clinical, lab, and CT data. **Special utility:** Prioritizing patients for bowel resection based on necrosis risk.
3D CNN-Based Obstruction Grading Model (Oh et al., 2023) (59)	Input: 3D CT images (non-contrast/contrast) Features: Automatically extracted radiomic features from entire CT volume	Deep learning model: 3D WideResNet backbone with dual-branch architecture + depth retention pooling. Training: Single-center retrospective dataset (*n* = 578; 250 normal, 209 HGSBO, 119 LGSBO) with 5-fold cross-validation.	Overall performance: Accuracy=72.6% AUROC=0.896 (95%CI: 0.895–0.897) Sensitivity=72.6%, Specificity=86.3%	Optimized architecture: DBA + DRP outperforms naive 3D CNN by 0.025 AUROC (0.896 vs. 0.861). Depth-aware: DRP preserves 3D spatial information critical for detecting focal lesions.	Single-center data, lacks external validation; “high-risk” labels based on imaging interpretation, not surgical gold standard.	Decision support for radiologists to automatically flag and grade high-risk obstruction cases on CT.
Multimodal Deep Learning Model (Wang, et al., 2023) (65)	1. DL CT prediction probability (extracted by 3D CNN ResNet50) 2. WBC count 3. CRP level 4. Abdominal rigidity 5. History of prior SBO episodes	AI model fusing deep learning image analysis (3D CNN) with clinical variables (XGBoost).	AUC=0.912 (Test Set) Sens 81%, Spec 92% Performance significantly better than single-modal models.	Excellent Performance: High AUC, good generalizability (multicenter validation). Interpretability: Provides basis for decisions.	Population-specific (Chinese cohort). Application Complexity: Requires corresponding technical deployment capability.	Precision risk assessment for ASBO patients with CT, to predict strangulation risk within 7 days and optimize timing of intervention.
Ischemia Prediction Score (IsPS) (Murao, et al., 2023) (40)	**s-IsPS (without CT):** WBC, Base Excess, Ascites. **m-IsPS (with CT):** adds CT decreased bowel wall enhancement.	Dual-version score for scenarios with/without contrast CT. Employs an objective CT enhancement threshold.	m-IsPS ≥3: Sens 87%, Spec 76%, AUC=0.84 s-IsPS used for screening when CT unavailable.	Clinical Versatility: Covers scenarios with and without CT. Imaging Objectivity: Employs explicit CT value threshold, reducing subjectivity.	Selection bias (excluded conservatively managed patients). s-IsPS uses base excess (requires arterial blood gas, not routine).	s-IsPS: Initial screening when contrast CT is not available/contraindicated. m-IsPS: Enhanced risk assessment when contrast CT is performed.
Imaging+Lab Multimodal AI Model (Vanderbecq et al., 2025) (49)	3D CT imaging features, CRP, Neutrophil count	Multimodal AI integrating a 3D CNN for CT, NLP for reports (FlauBERT), and an MLP for lab data.	External Test (Image + Lab): AUC=0.69, Sens=0.89, Spec=0.44. Improved physician diagnosis.	Multimodal Advantage: Fuses imaging and laboratory data. Clinical Aid Value: Reduces interpretation variability. Focus on Early Decision: Designed to support critical decisions within 24 h of admission.	Poor generalization of the model using radiology report text. Gold standard limitation: based on surgical findings within 24 h, may miss delayed ischemia.	Clinical decision support for early (within 24 h of admission) risk assessment, particularly to assist less experienced clinicians.

SIRS, systemic inflammatory response syndrome; I-FABP, intestinal fatty acid-binding protein; BAR-N, BMI-AST-rebound tenderness-neutrophil percentage model; CT, computed tomography; HU, hounsfield unit; AUC, area under the curve; Sens, sensitivity; Spec, specificity; OR, odds ratio; RS, risk score; AI, artificial intelligence; CNN, convolutional neural network; NLP, natural language processing; MLP, multilayer perceptron; IsPS, Ischemia prediction score; m-IsPS, modified Ischemia prediction score; ASBO, adhesive small bowel obstruction; ABS, Antoine-Béclère score; CRP, C-reactive protein; WBC, white blood cell; BUN, blood urea nitrogen; AP, anteroposterior.

a SIRS criteria: Temperature >38 °C or <36 °C; Heart rate >90 bpm; Respiratory rate >20/min or PaCO₂ < 32 mmHg; WBC >12,000/µL or <4,000/µL or >10% immature bands.

One particular category emphasizes the integration of clinical symptoms and laboratory indices for practical use in early emergency settings. Maraux et al. (47), based on retrospective (*n* = 171) and prospective (*n* = 72) cohorts, proposed the Antoine-Béclère Score (ABS), which incorporates three readily accessible admission variables: an age-adjusted Charlson Comorbidity Index of ≥4, CT-determined distal (ileal) obstruction, and a small bowel maximal diameter to abdominal wall diameter ratio of >0.34.A score ranging from 0 to 3 indicates a strangulation risk between 21% and 70%. The innovation of this model lies in the introduction of the bowel diameter/abdominal diameter ratio, a morpho-functional composite index that considers individual variations in abdominal cavity volume. All parameters can be acquired without the use of oral contrast within a 10-minute timeframe, facilitating rapid triage upon patient intake in emergency departments. Nonetheless, this model was developed at a single French center and has undergone limited external validation. Its capacity to differentiate the gray zone between early reversible ischemia and irreversible necrosis is inadequate. In contrast, Zheng et al. (21) employed LASSO regression on 22 candidate variables to identify BMI, AST, rebound tenderness, and neutrophil percentage as components of the BAR-N score. This model is parsimonious and easily applicable, making it particularly advantageous in resource-constrained emergency settings. However, its sensitivity is generally low (<40%), rendering it more effective for ruling out strangulation rather than for early detection. Additionally, the absence of imaging data restricts its overall informational value.

Through comprehensive investigation, researchers have progressively enhanced the integration of imaging parameters by developing static models focused on radiological findings. CT scoring systems, based on multi-detector CT indicators such as bowel wall thickening, mesenteric fluid, and decreased wall enhancement, are designed to accurately differentiate transmural necrosis from reversible ischemia (45). For example, Chai et al. (48) incorporated subjective CT indicators (e.g., closed-loop, decreased wall enhancement, mesenteric edema) with conventional Hounsfield Unit (HU) measurements, proposing dual-threshold criteria (enhancement value <21.5 HU or pre-contrast to post-contrast difference >16.5 HU) to achieve 100% specificity in diagnosing necrosis, with an area under the curve (AUC) of 0.925. This model captures only instantaneous imaging-physiological information at the time of CT scanning. Consequently, it lacks dynamic weighting updates for the continuum between reversible ischemia and necrosis, resulting in insufficient sensitivity for early ischemia. By contrast, a multicenter study by Vanderbecq et al. (49) employed artificial intelligence algorithms to process the entire abdominal-pelvic CT 3D matrix as input for autonomous learning of “strangulation” features. Subsequently, fusing these with static admission data (CRP, neutrophils). While external validation yielded an AUC of 0.69, it pioneered a new high-dimensional feature space for multiparametric static modeling in bowel obstruction.

Subsequently, these were integrated with static admission data, including C-reactive protein (CRP) and neutrophil counts. Kudou et al. (50) developed an effective predictive scoring system for risk stratification by combining ascites, computed tomography (CT) values, lactate levels, and the neutrophil-to-lymphocyte ratio (NLR). Huang et al. (39) incorporated five indicators—body temperature ≥38.0 °C, peritoneal signs, white blood cell count >10 × 10^9/L, CT bowel wall thickening ≥3 mm, and ascites—achieving an AUC of 0.935, which indicates excellent discriminatory ability. However, the evaluation of peritoneal signs is contingent upon clinical expertise, and inter-observer variability (*κ*=0.48) diminishes reproducibility. Additionally, score thresholds may vary considerably with repeated measurements at different time points. A multicenter prospective study identified seven independent risk factors: pain duration of ≤3 Days, rebound tenderness, diminished or absent bowel sounds, hypokalemia, hyponatremia, elevated blood urea nitrogen (40). The developed multidimensional model demonstrated excellent performance in identifying patients with small bowel obstruction (SBO) who required surgical intervention (AUC 0.857). It also accurately identified individuals with intestinal perforation or necrosis, with a significantly higher proportion observed in the high-risk group. However, the model predominantly relies on static baseline data obtained at initial admission, thereby overlooking dynamic changes in laboratory or imaging findings during the observation period. Additionally, the external validation was conducted on a relatively small sample size (*n* = 123), highlighting the need for larger population studies to confirm the model's stability (51).

Recent investigations have begun exploring the inclusion of novel composite inflammatory indices. The lymphocyte-to-monocyte ratio (LMR) and systemic inflammation index (SII) have been associated with clinical outcomes in simple intestinal obstruction (OR=0.656). However, within the SSBO subgroup, only blood urea nitrogen (OR=1.478) and lymphocyte count (OR=0.071) demonstrated independent predictive value (52). Murao et al. (12) introduced the Ischemia Prediction Score (IsPS), which initially included variables such as white blood cell count, low alkaline phosphatase levels, ascites, and reduced bowel wall enhancement. A modified version, the mIsPS, demonstrated a sensitivity of 86.7% and a specificity of 76.0% for predicting intestinal ischemia when using a cut-off score of ≥3 points. Although these investigations expand the range of potential risk factors, the absence of standardized protocols for development and validation hampers the comparability of different models, and their clinical utility has yet to be established.

In conclusion, multiparametric static models represent a notable advancement in risk stratification by improving predictive accuracy and stability through comprehensive evaluation. However, they exhibit common limitations. These models are typically based on data collected at a single, specific time point (usually at initial admission), which fails to account for the dynamic and evolving nature of small bowel obstruction (SBO) pathophysiology, where a simple obstruction can rapidly progress to strangulation. Additionally, external validation is generally insufficient. The robustness of these models must be rigorously demonstrated through independent validation across diverse geographical regions, timeframes, and patient populations. At present, the majority of models discussed in the literature have only been subjected to internal validation methods, such as cross-validation or bootstrapping, which are insufficient to establish their widespread clinical applicability. Therefore, caution is advised when considering their application in clinical settings.

## 4 Towards dynamism and intelligence: methodologies and technical pathways for multiparametric risk modeling

### 4.1 Technological foundation: AI algorithms and multimodal data fusion

The development of dynamic multiparametric models is contingent upon the ability of artificial intelligence (AI) algorithms to effectively integrate and analyze multimodal, heterogeneous healthcare data. In the field of medical image processing, deep learning models—particularly Convolutional Neural Networks (CNNs) and their three-dimensional extensions (3D-CNNs)—have emerged as pivotal tools for extracting high-dimensional radiomic features (53, 54). These models are capable of automatically segmenting and extracting features from abdominal CT scans, identifying subtle patterns that are not discernible to the human eye but are indicative of conditions such as bowel wall ischemia or necrosis. These patterns include mural stratification, enhancement heterogeneity, and subtle mesenteric changes (55, 56).

For example, Kim et al. (57) developed an ensemble CNN model capable of detecting small bowel obstruction on plain abdominal radiographs, achieving an area under the curve (AUC) of 0.961, with a sensitivity of 91% and specificity of 93%. Similarly, Cui et al. (58) utilized ResNet and DenseNet architectures, incorporating ImageNet pre-trained weights and the Adam optimizer within a ten-fold cross-validation framework, to model the early diagnosis of neonatal necrotizing enterocolitis (NEC). This approach effectively enhanced the model's generalizability on small-sample medical data. These approaches effectively compress high-dimensional imaging data into interpretable feature vectors, thereby establishing a foundation for subsequent multimodal fusion with other data modalities, such as time-series vital signs. External validation indicates that such multimodal models achieve diagnostic performance (AUC=0.83) comparable to that of experienced pediatric radiologists (AUC=0.82), highlighting their significant potential as clinical assistive tools.

Additionally, a research team from Ajou University in South Korea developed a deep learning model based on 38,000 CT images (59). They employed a dual-branch architecture (DBA) combined with a depth-retained pooling (DRP) strategy. The DBA effectively decouples the classification task by employing a base classifier to differentiate between normal, low-risk, and high-risk acute small bowel obstruction. Concurrently, a fine classifier is utilized to discern subtle distinctions between high-risk and low-risk cases, thereby enhancing the model's capacity to differentiate similar pathological features through simultaneous training. The Depth Retention Pooling (DRP) mechanism plays a crucial role in preserving essential slice information along the depth dimension of CT scans, thus mitigating the loss of subtle lesion information typically associated with conventional pooling operations. The study further substantiated the model's robustness and interpretability by simulating low-quality images (e.g., blurring, contrast abnormalities) and employing Grad-CAM visualization, ultimately achieving precise stratification of high-risk vs. low-risk patients.

This provides objective decision support for emergency triage. For processing time-series data, such as vital signs and laboratory values, Recurrent Neural Networks (RNNs) and their advanced variants, including Long Short-Term Memory networks (LSTMs) and Gated Recurrent Units (GRUs), are widely utilized (60). Recently, temporal convolutional networks (TCNs) have shown promising performance in clinical time-series prediction and, in some settings, have outperformed recurrent neural networks in modeling longitudinal data for early risk prediction (61). In the context of dynamic SBO risk assessment, TCNs can efficiently process continuously monitored data (over hours to days) of parameters like WBC count, serum lactate, and inflammatory markers, precisely quantifying their trajectories, rates of change, and variability to identify dynamic patterns predictive of clinical deterioration. Ultimately, through advanced fusion strategies (e.g., attention-based fusion, multimodal neural networks), feature vectors extracted from multi-source information—imaging, time-series vitals/labs, and static baseline characteristics—can be integrated to construct a unified model framework aimed at outputting a dynamic, comprehensive risk assessment.

### 4.2 Core paradigm: from static snapshot to dynamic continuous risk assessment

The core paradigm shift lies in moving from a static snapshot to a dynamic, continuous assessment of disease evolution, underpinned by two pillars: dynamism and deep multiparametric integration. It necessitates not only the inclusion of baseline data upon admission but also the continuous acquisition and analysis of trends in various indicators over time. For laboratory markers like serum lactate or CRP, the rate of rise, fluctuation patterns, and dynamic thresholds may hold greater predictive significance than isolated values (17). Methodologically, time-series algorithms (e.g., LSTM, TCN) can learn the complex relationships between these dynamic patterns and final clinical outcomes, outputting a continuously updated risk probability curve as monitoring progresses (62). The Bio-Shift model developed by Klimczak-Tomaniak et al. (63) for chronic heart failure provides a pertinent example.By constructing a temporal panel comprising 92 protein biomarkers measured at the three-month mark, utilizing LASSO penalized regression for high-dimensional feature selection, and employing a multivariable joint model to directly associate individualized biomarker trajectories with time-to-event outcomes, the researchers achieved dynamic risk estimation updates with the incorporation of new data. Their model exhibited superior performance (cross-validated AUC=0.88) compared to static baseline models, highlighting the importance of temporal integration in capturing the continuous evolution of pathophysiological processes.

Nevertheless, it is imperative to maintain methodological rigor. A systematic review of real-time sepsis prediction models indicated that only 54.9% of studies implemented full-window validation, resulting in 78.3% of models demonstrating negative utility scores in external cohorts, thereby revealing a deficiency in clinical translation potential (64). This finding underscores a critical warning for dynamic SBO prediction: high internal AUC values do not guarantee clinical usefulness once deployed in heterogeneous external environments. External validation remains insufficient in many AI studies, and failures frequently reflect poor generalization across institutions, differing case mixes, and variable practice patterns. In addition, real-world applicability is frequently challenged by data heterogeneity, missingness, and inconsistent sampling frequencies. Distributional shifts—caused by evolving disease spectra, changes in imaging protocols, laboratory assay updates, or modifications in clinical pathways—can lead to data drift and performance decay over time. Without continuous monitoring and recalibration, model predictions may gradually diverge from reality.

This underscores the critical need, when developing dynamic predictive models for SSBO, to establish intensive monitoring systems (e.g., with hourly resolution), convert biomarkers such as I-FABP and hemodynamic responses into continuous time-series data, and employ temporal algorithms to identify dynamic patterns associated with ischemic events. Imaging parameters require the extraction of both quantitative and qualitative features from modalities like CT and ultrasound, which also include radiomic features. Additionally, attention must be paid to treatment response parameters, specifically the patient's response to initial conservative measures such as nasogastric decompression and fluid resuscitation. Effective integration requires addressing challenges like data heterogeneity, missingness, and inconsistent sampling frequencies across parameters. This is tackled through feature selection or embedding methods to identify the most predictive feature combinations (65).

### 4.3 Clinical translation: from theoretical models to clinical tools

The construction of dynamic multi-parametric models can be conceptualized as a systematic, multi-stage methodology. The foundational step involves building a robust data infrastructure by establishing an integrated data architecture. This requires breaking down silos between Hospital Information Systems (HIS), Laboratory Information Systems (LIS), Picture Archiving and Communication Systems (PACS), and intensive care unit (ICU) monitoring systems to enable multi-source data interoperability. The goal is to aggregate these streams into standardized, longitudinal patient data repositories, providing the essential fuel for model training and real-time analytics. Building upon this foundation, meticulous feature engineering and representation learning are performed. Static features undergo standardized encoding, while for temporal features, algorithms are employed to automatically extract key statistical properties (e.g., mean, variance, trends), construct sliding-window features, or leverage raw sequential data for end-to-end deep learning, thereby comprehensively capturing underlying temporal dependencies (66).

The subsequent core phase involves algorithm selection and model training, tailored to the data characteristics and the specific predictive task—be it classification, regression, or time-to-event analysis. To ensure a rigorous and unbiased evaluation of model performance and prevent data leakage, methodologies such as temporal cross-validation must be strictly employed. Model assessment must then extend beyond conventional static metrics. While discriminative power, measured by indices like the Area Under the Receiver Operating Characteristic Curve (AUC), is important, equal emphasis should be placed on temporal predictive accuracy, calibration (the agreement between predicted probabilities and observed outcomes), and clinical net benefit as evaluated by Decision Curve Analysis.

To ensure sustained effectiveness in long-term clinical deployment, proactive mechanisms to address “model drift” are imperative. Drift, or performance decay over time, can result from evolving disease spectra, updates in clinical practice, or advancements in detection technology (67). Dynamic models must therefore incorporate continuous learning and self-updating capabilities, ranging from foundational periodic retraining with recent data to more advanced approaches like online learning for real-time, incremental updates. In multi-center settings, Federated Learning frameworks enable collaborative, distributed model optimization across institutions while rigorously preserving data privacy, fostering a continuously evolving medical AI ecosystem.

However, successful translation also requires confronting regulatory and governance challenges. High-dimensional black-box models complicate transparency requirements for medical device approval, and insufficient explainability may hinder clinician acceptance. Prospective multicenter validation remains limited, and few studies have demonstrated improved patient-centered outcomes or cost-effectiveness in real-world trials. Importantly, the absence of standardized outcome definitions and labeling criteria further complicates cross-study comparability.

Ultimately, successful clinical translation hinges on seamless integration into the clinical workflow as a trustworthy decision-support tool. This involves embedding predictive outputs—presented via intuitive visualizations like risk dashboards or trend curves—directly into the Electronic Health Record (EHR) and configuring automated alerts for critical junctures. Human-AI collaboration is strengthened by enhancing model explainability using techniques such as SHAP or LIME to transparently reveal the rationale behind predictions, empowering clinicians to interpret rather than blindly follow AI suggestions. Finally, prospective validation of clinical efficacy and health economic evaluation through rigorously designed studies (e.g., randomized controlled trials, stepped-wedge cluster designs) is essential to demonstrate the model's real-world impact on patient outcomes and healthcare resource utilization, thereby conclusively proving its clinical value and cost-effectiveness. At the current stage, AI-based dynamic risk models should be regarded as assistive tools rather than autonomous decision-makers; they remain insufficient to replace clinical judgment.

## 5 Future research directions

The potential implementation of precision triage for SSBO will likely require the synergistic integration of “mechanism, data, and decision-making.” This integration is manifested through the advancement of basic research, innovation in technical paradigms, and systematic optimization of clinical translation pathways. Basic research must extend beyond conventional inflammatory markers to explore specific molecular pathways of cell death. Recently, novel mechanisms of programmed cell death have gained attention in the context of ischemia-reperfusion injury (68). Ferroptosis, an iron-dependent form of cell death driven by lipid peroxidation, shows promise. The activation of its key regulatory proteins may directly indicate the extent of oxidative stress damage in intestinal cells, presenting potential new targets for early diagnosis (69). Tissue engineering offers promising avenues for bowel repair and regeneration. Studies employing decellularized matrix scaffolds, such as small intestinal submucosa or collagen sponges in conjunction with intestinal organoids or mesenchymal stem cells, have successfully developed engineered intestinal tissue with partial functionality (70, 71). Nonetheless, this field is still in its nascent stages and faces numerous challenges.

At the data and technology level, systemic interoperability and real-time data processing are significant bottlenecks. Dynamic risk stratification models necessitate real-time, automated data extraction and standardization from hospital IT systems. Presently, many information systems are hindered by legacy architectures and inconsistent data standards, rendering the construction of unified data streams exceedingly difficult (72). Additionally, real-time model deployment and computation require a robust IT infrastructure. Moreover, since dynamic models may qualify as Class II or higher-risk Software as a Medical Device (SaMD), AI-assisted diagnostic systems must undergo rigorous clinical validation and obtain regulatory approval (e.g., from the FDA or CE) prior to clinical implementation (73).

In the realm of Clinical Translation and Validation, a significant limitation of current research is the absence of large-scale, prospective, multi-center external validation for the majority of models, which considerably hampers their generalizability. Future research efforts should prioritize conducting prospective multi-center validation studies (74, 75). Trial designs must delineate clear primary and secondary endpoints, with diagnostic sensitivity and specificity serving as key primary endpoints (76). Studies should incorporate predefined, objective criteria for patient inclusion and exclusion, employ scientific sample size calculations, and focus on hard clinical endpoints. It is crucial to involve clinicians and regulatory scientists from the early stages of development to proactively manage inter-center heterogeneity—such as through standardized CT protocols and clinical pathways—and systematically assess its impact on model performance (17). Furthermore, fostering the deep integration of models into clinical workflows is imperative. This can be achieved by embedding them into Electronic Health Records (EHRs) and developing them into Clinical Decision Support Systems (CDSS) that automatically retrieve data and provide instantaneous risk assessments and decision support, thereby enabling dynamic multiparametric models to effectively bridge existing gaps.

## 6 Conclusion

SSBO is a high-acuity surgical emergency. Its management is challenging due to a mismatch between the dynamically evolving ischemia-inflammatory cascade and conventional static assessment systems. Traditional methods, which rely on isolated indicators, are inadequate for capturing the continuous progression from reversible ischemia to irreversible necrosis and fail to effectively balance the risks of “overtreatment” against “delayed intervention”. The field of SSBO assessment is currently experiencing a paradigm shift from static, single-dimension approaches to dynamic, intelligent systems. This review highlights that intestinal ischemia is a continuous process involving local initiation, inflammatory amplification, and systemic progression, which necessitates a focus on temporal trends rather than single-time-point data. Dynamic multiparametric models, by monitoring the evolution of key indicators, hold promise for earlier risk identification and enable real-time, personalized assessments.

Recent advancements in artificial intelligence and machine learning algorithms suggest a foundational capability for processing multimodal, temporal medical data. While AI enables the integration of multimodal data into dynamic, personalized risk curves, most current models remain in the developmental stage and lack robust clinical validation across diverse populations. Furthermore, challenges related to data system interoperability and workflow integration present substantial obstacles to achieving genuine clinical translation. Developing an effective precision triage system for SSBO necessitates multidimensional collaboration across the domains of mechanism, data, technology, and clinical practice.
